# Supplementary material for: Transfer learning reveals sequence determinants of the quantitative response to transcription factor dosage
Source: bioRxiv. 2024 May 29:2024.05.28.596078. Preprint. [Version 1] doi: 10.1101/2024.05.28.596078 (PMC11160683; doi:10.1101/2024.05.28.596078)
Supplement: Supplement 7 [file NIHPP2024.05.28.596078v1-supplement-7.pdf]

bioRxiv preprint doi: https://doi.org/10.1101/2024.05.28.596078; this version posted May 29, 2024. The copyright holder for this preprint (which was not certified by peer review) is the author/funder, who has granted bioRxiv a license to display the preprint in perpetuity. It is made available under aCC-BY 4.0 International license.

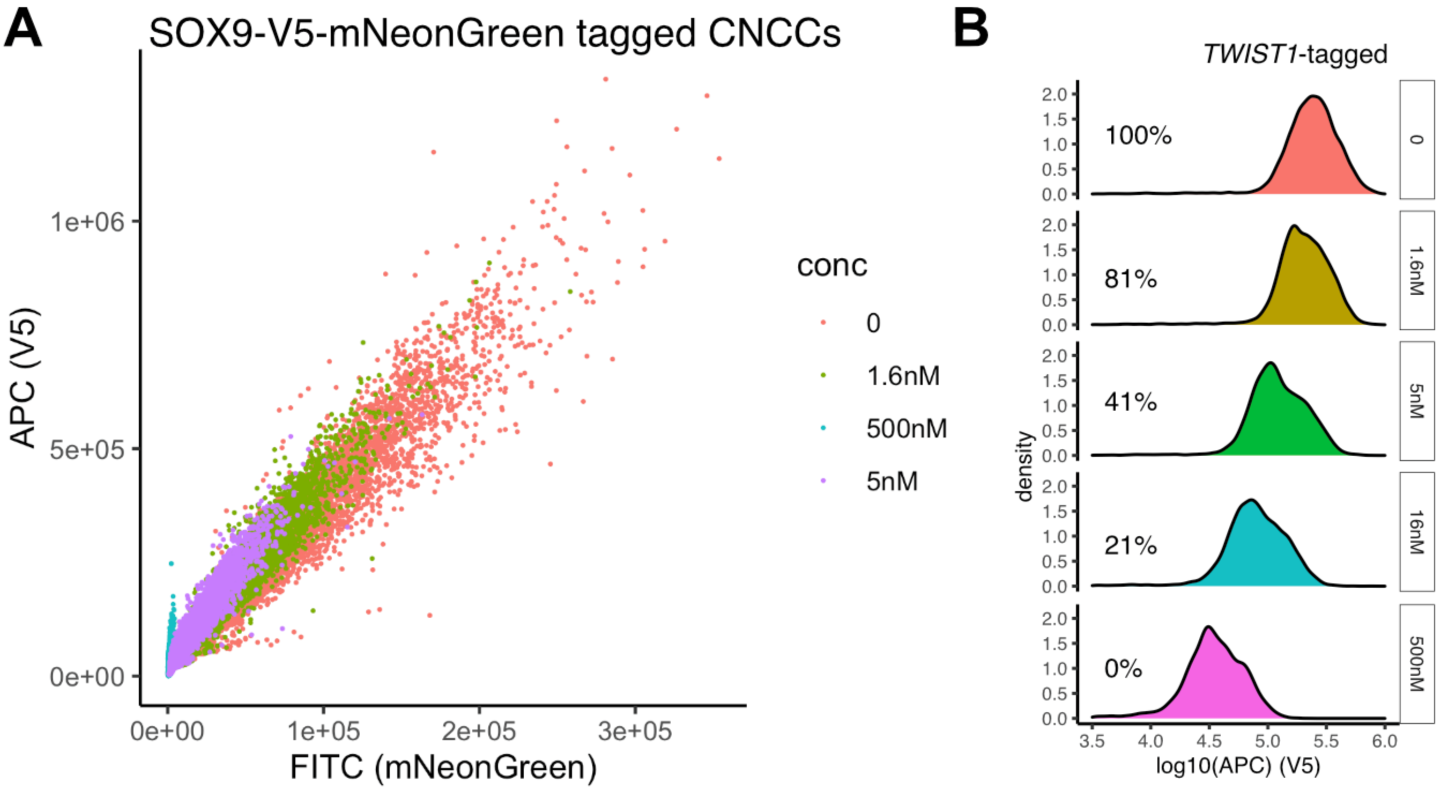

Figure S1. Precise modulation of TWIST1 dosage. (A) Comparison of V5 (y-axis) and mNeonGreen (x-axis) signal in single SOX9-tagged cells treated with different dTAGV-1 concentrations. (B) Second independent replicate of TWIST1 dosage modulation, as in Figure 1B.

bioRxiv preprint doi: https://doi.org/10.1101/2024.05.28.596078; this version posted May 29, 2024. The copyright holder for this preprint (which was not certified by peer review) is the author/funder, who has granted bioRxiv a license to display the preprint in perpetuity. It is made available under aCC-BY 4.0 International license.

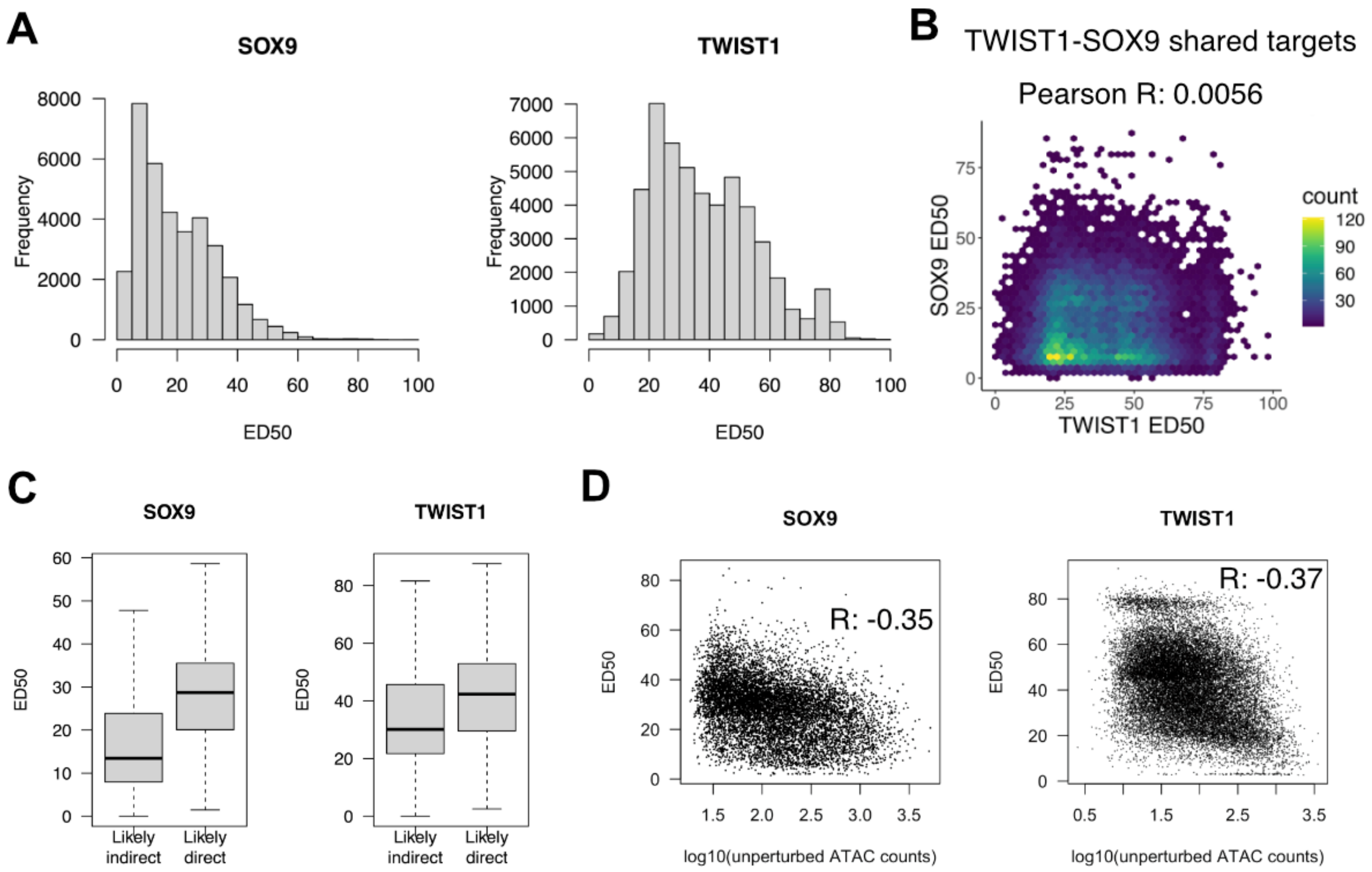

Figure S2. RE sensitivity to SOX9 and TWIST1 dosage. (A) Distribution of ED50 values among all SOX9 or TWIST1-dependent REs. (B) ED50 with respect to SOX9 dosage (y-axis) and TWIST1 dosage (x-axis) for all REs that are both SOX9- and TWIST1-dependent. (C) ED50 of likely direct or indirect SOX9 or TWIST1 targets. For SOX9, likely direct targets were defined as the 3h downregulated class as in Naqvi et al 2023, and for TWIST1, direct targets were defined as downregulated and containing a TWIST1 ChIP-seq peak. (D) Unperturbed accessibility (x-axis) versus ED50 (y-axis) for all likely direct SOX9 or TWIST1 targets.

bioRxiv preprint doi: https://doi.org/10.1101/2024.05.28.596078; this version posted May 29, 2024. The copyright holder for this preprint (which was not certified by peer review) is the author/funder, who has granted bioRxiv a license to display the preprint in perpetuity. It is made available under aCC-BY 4.0 International license.

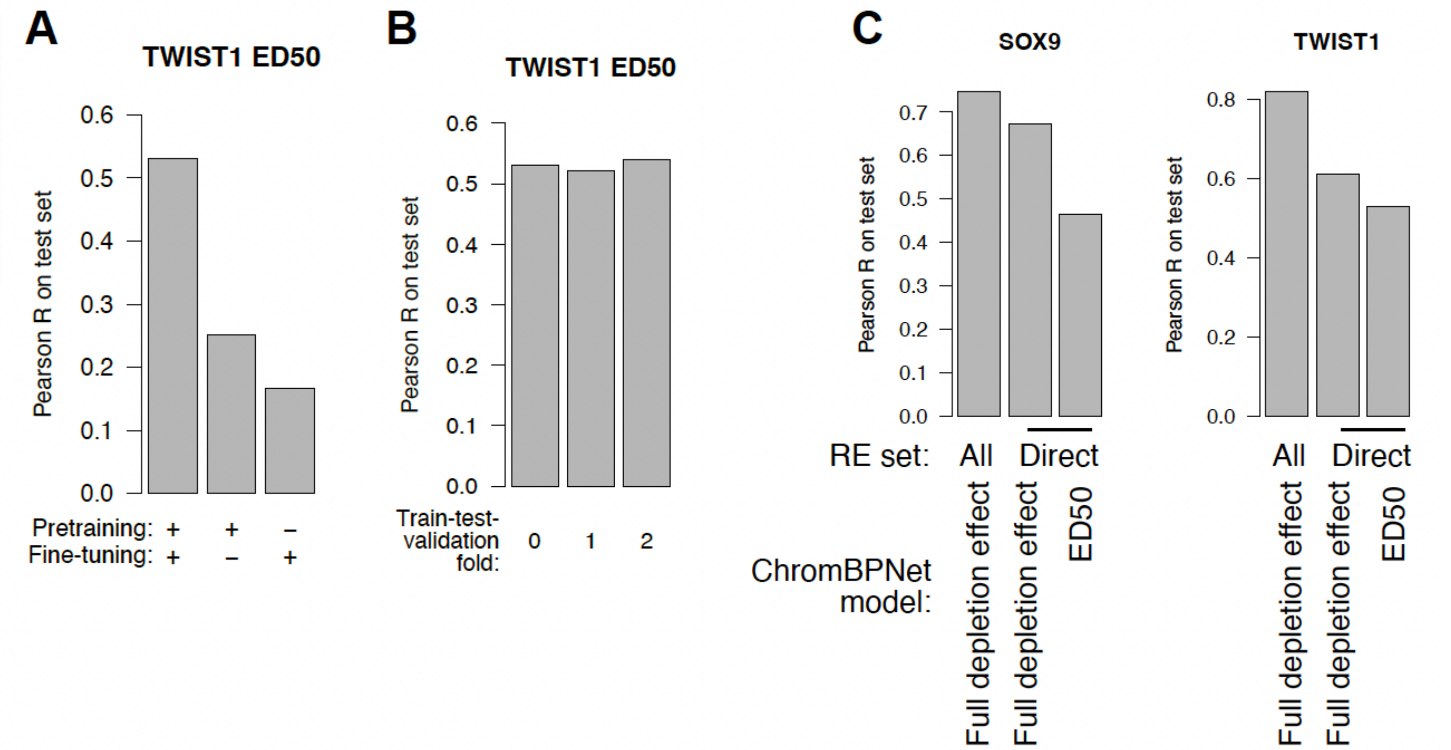

Figure S3. Prediction of effect of full TF depletion and ED50 from DNA sequence. (A) Prediction accuracy of TWIST ED50 prediction with and without pretraining or fine-tuning of ChromBPNet model. (B) Performance of pretrained and fine-tuned ChromBPNet model for predicting TWIST1 ED50 across three independent train-test-validation splits. (C) Decreased performance of ChromBPNet model for prediction effect of full SOX9 (left) or TWIST1 (right) depletion when predictions are subsetted to only direct RE targets (middle bar in each plot).

bioRxiv preprint doi: https://doi.org/10.1101/2024.05.28.596078; this version posted May 29, 2024. The copyright holder for this preprint (which was not certified by peer review) is the author/funder, who has granted bioRxiv a license to display the preprint in perpetuity. It is made available under aCC-BY 4.0 International license.

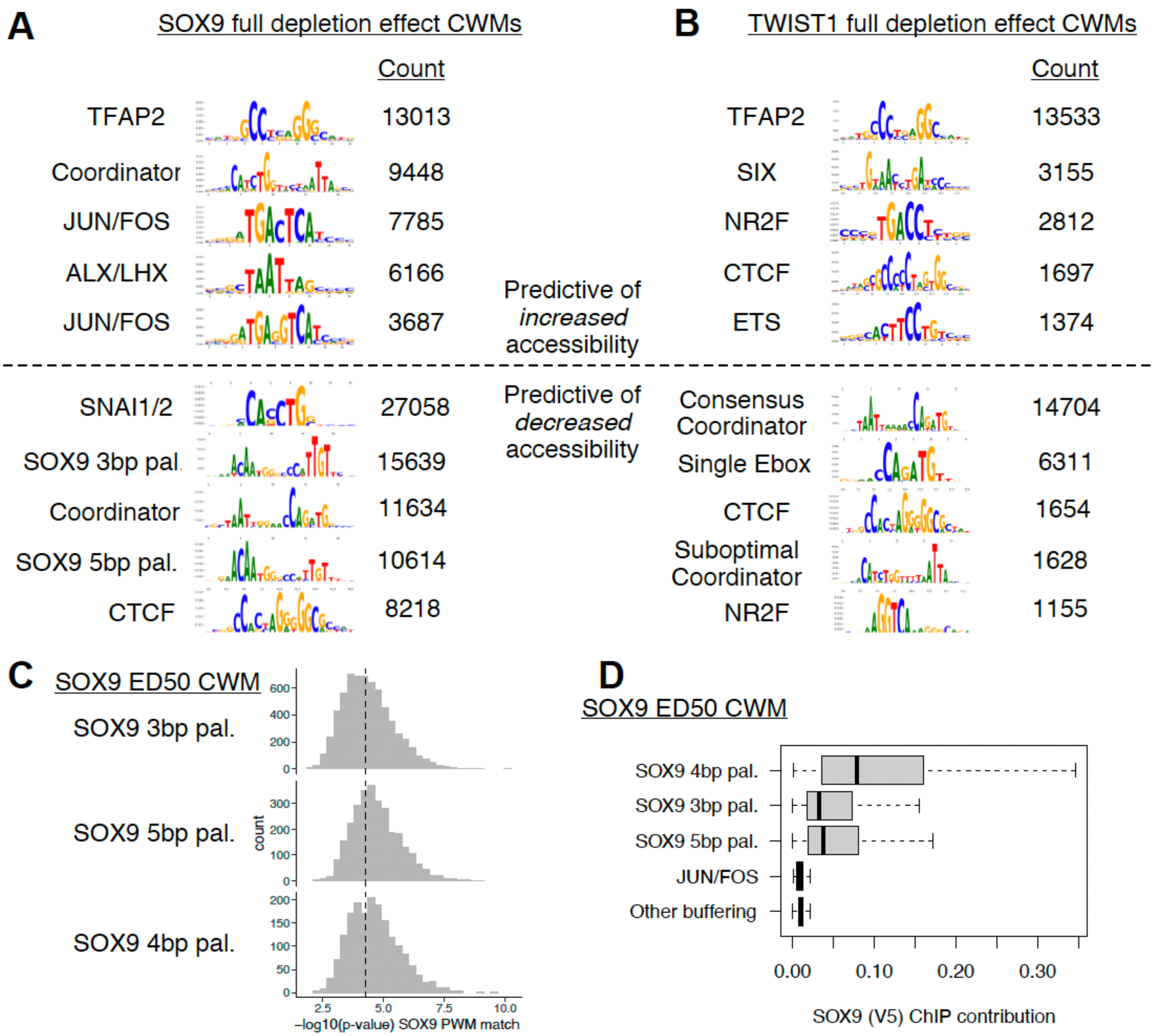

Figure S4. Sequence features predictive of the effect of full TF depletion on RE accessibility. (A,B) Top contribution weight matrices (CWMs) predictive of effect of full depletion of (A) SOX9 or (B) TWIST1 on RE accessibility. Number of individual occurrences of each CWM is indicated under the "count" column. (C) For all individual instances of the indicated CWMs predictive of SOX9 ED50 (rows), the strength of that sequence match to SOX9 palindrome position weight matrix (PWM) is shown (x-axis). (D) For the indicated CWMs, the distribution of contribution to SOX9 binding, estimated from BPNet on SOX9-V5 ChIP-seq, is shown.

bioRxiv preprint doi: https://doi.org/10.1101/2024.05.28.596078; this version posted May 29, 2024. The copyright holder for this preprint (which was not certified by peer review) is the author/funder, who has granted bioRxiv a license to display the preprint in perpetuity. It is made available under aCC-BY 4.0 International license.

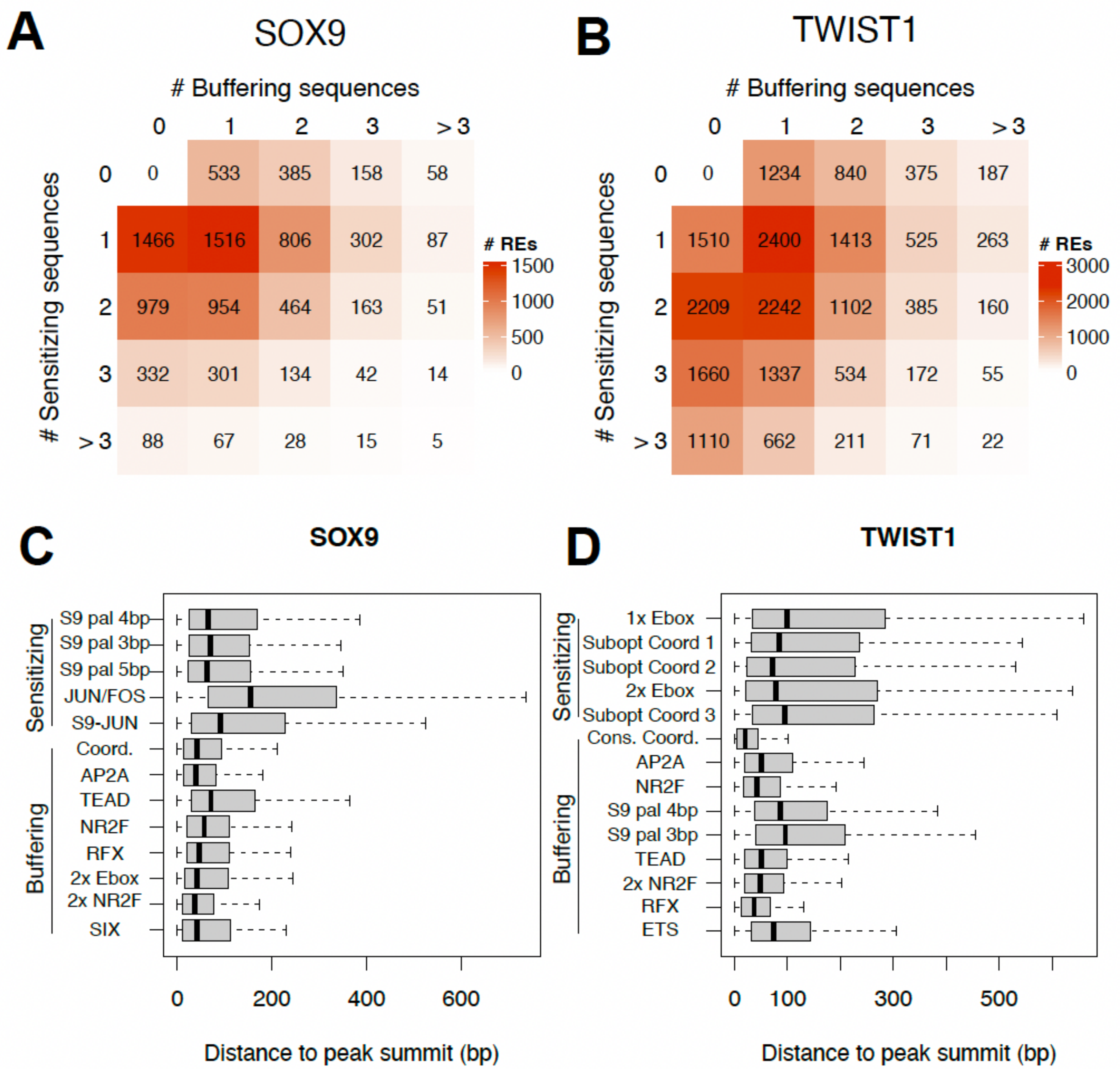

Figure S5. Additional features of buffering and sensitizing sequences. (A,B) The number of (A) SOX9 or (B) TWIST1 target REs with the indicated number of buffering (x-axis) or sensitizing (y-axis) CWM occurrences. (C,D), Distance to ATAC peak summit of individual types of sensitizing or buffering CWM occurrences for SOX9 (C) or TWIST1 (D).

bioRxiv preprint doi: https://doi.org/10.1101/2024.05.28.596078; this version posted May 29, 2024. The copyright holder for this preprint (which was not certified by peer review) is the author/funder, who has granted bioRxiv a license to display the preprint in perpetuity. It is made available under aCC-BY 4.0 International license.

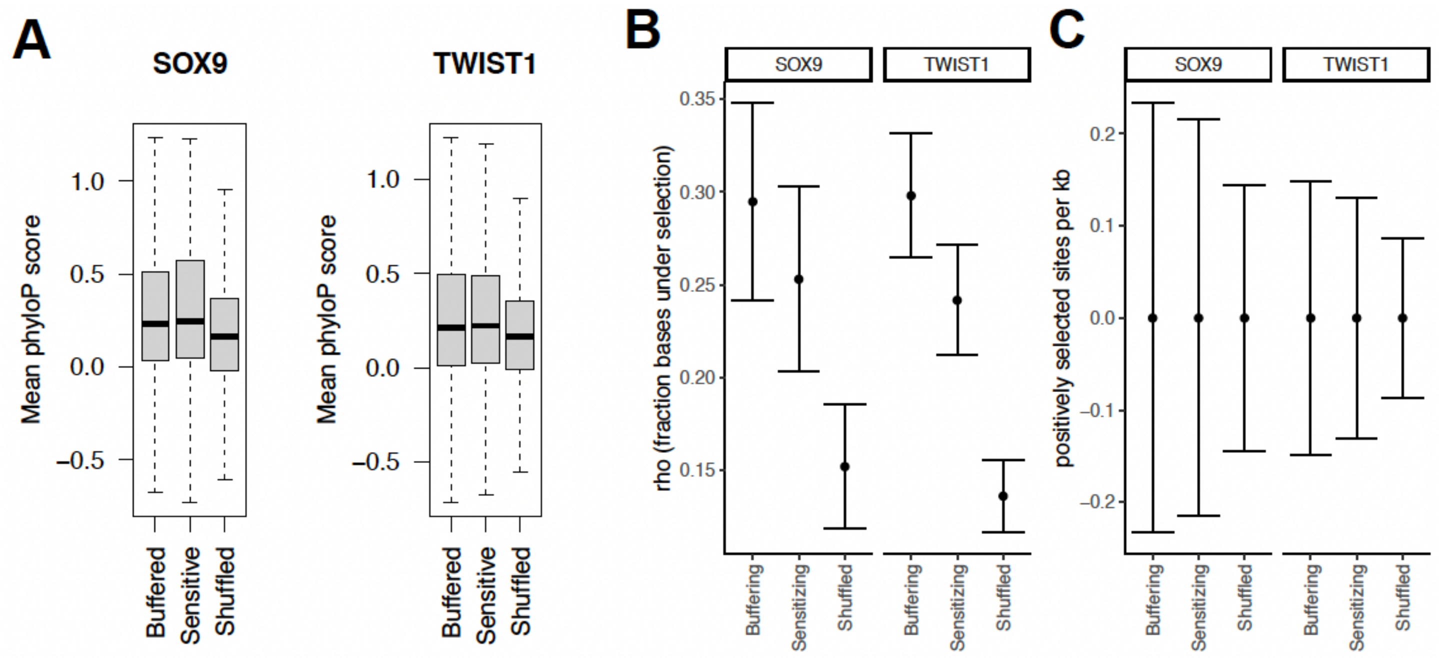

Figure S6. Signatures of selection at sensitizing and buffering sequences. (A) Mean phyloP score of buffering, sensitizing, or shuffled motif occurences for SOX9 or TWIST1 ED50. Positive phyloP scores mean more likely to be conserved, negative means more likely to be under positive selection. (B,C) Fraction of sites under weak negative selection (B) or frequency of sites under positive selection (C) for the same classes of motifs as in (A), estimated by INSIGHT.

bioRxiv preprint doi: https://doi.org/10.1101/2024.05.28.596078; this version posted May 29, 2024. The copyright holder for this preprint (which was not certified by peer review) is the author/funder, who has granted bioRxiv a license to display the preprint in perpetuity. It is made available under aCC-BY 4.0 International license.

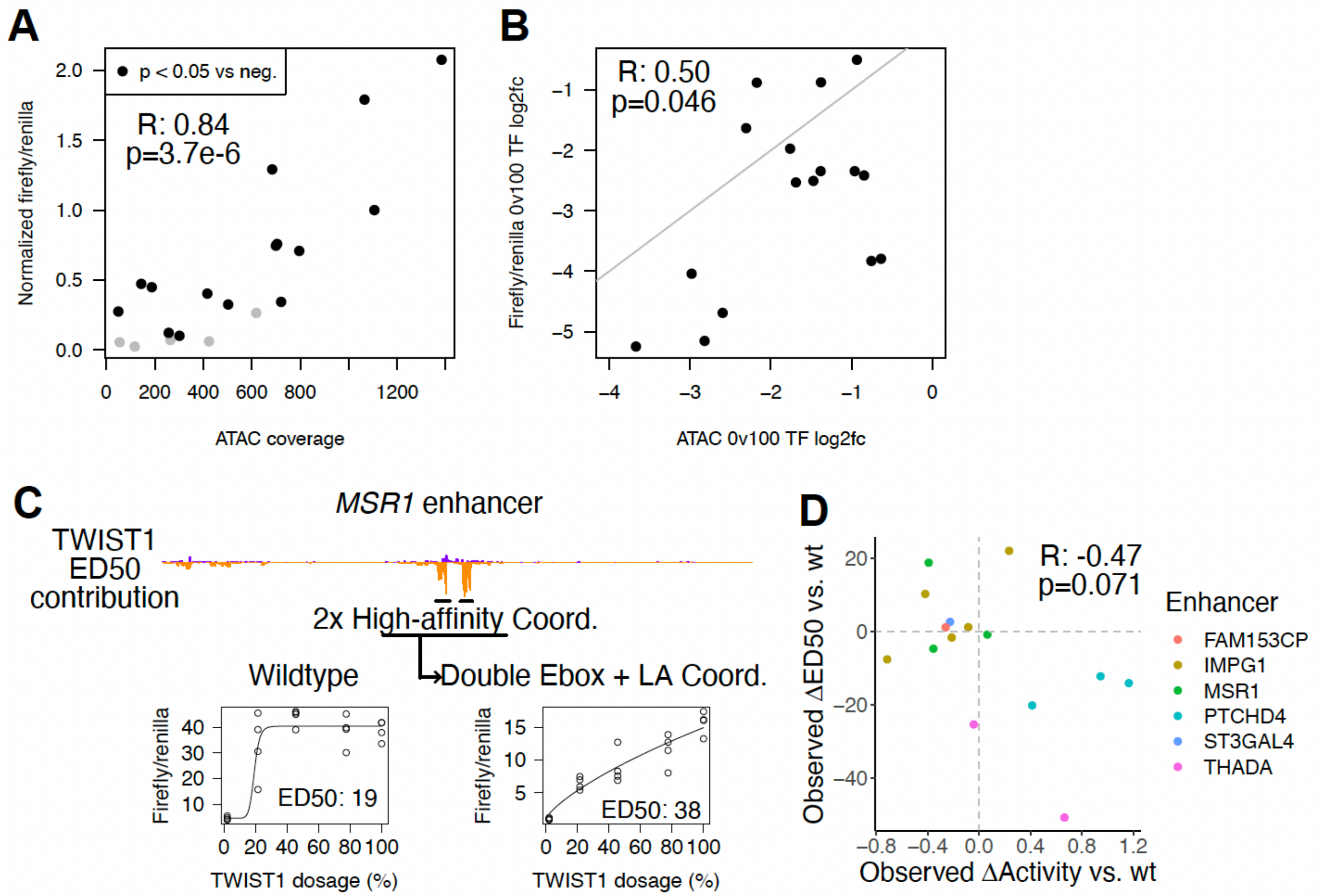

Figure S7. Dosage responses of wildtype and mutant REs in enhancer reporter assays. (A) Comparison of endogenous unperturbed accessibility (x-axis) and enhancer reporter activity (y-axis, normalized to positive control in each experiment) across 19 TWIST1- and SOX9-dependent REs (points). (B) For the REs in (A) with significantly higher activity than the negative control, the effect of full TF depletion on endogenous accessibility (x-axis) is compared to the effect of full TF depletion on enhancer reporter activity (y-axis). (C) Example of *MSR1* enhancer, where converting two high-affinity, buffering Coordinator motifs into a double E-box and low-affinity (LA) Coordinator motif has a sensitizing effect. (D) Comparison of changes relative to wildtype in reporter activity at 100% TF dosage (x-axis) and ED50 (y-axis) for tested mutant enhancers.

bioRxiv preprint doi: https://doi.org/10.1101/2024.05.28.596078; this version posted May 29, 2024. The copyright holder for this preprint (which was not certified by peer review) is the author/funder, who has granted bioRxiv a license to display the preprint in perpetuity. It is made available under aCC-BY 4.0 International license.

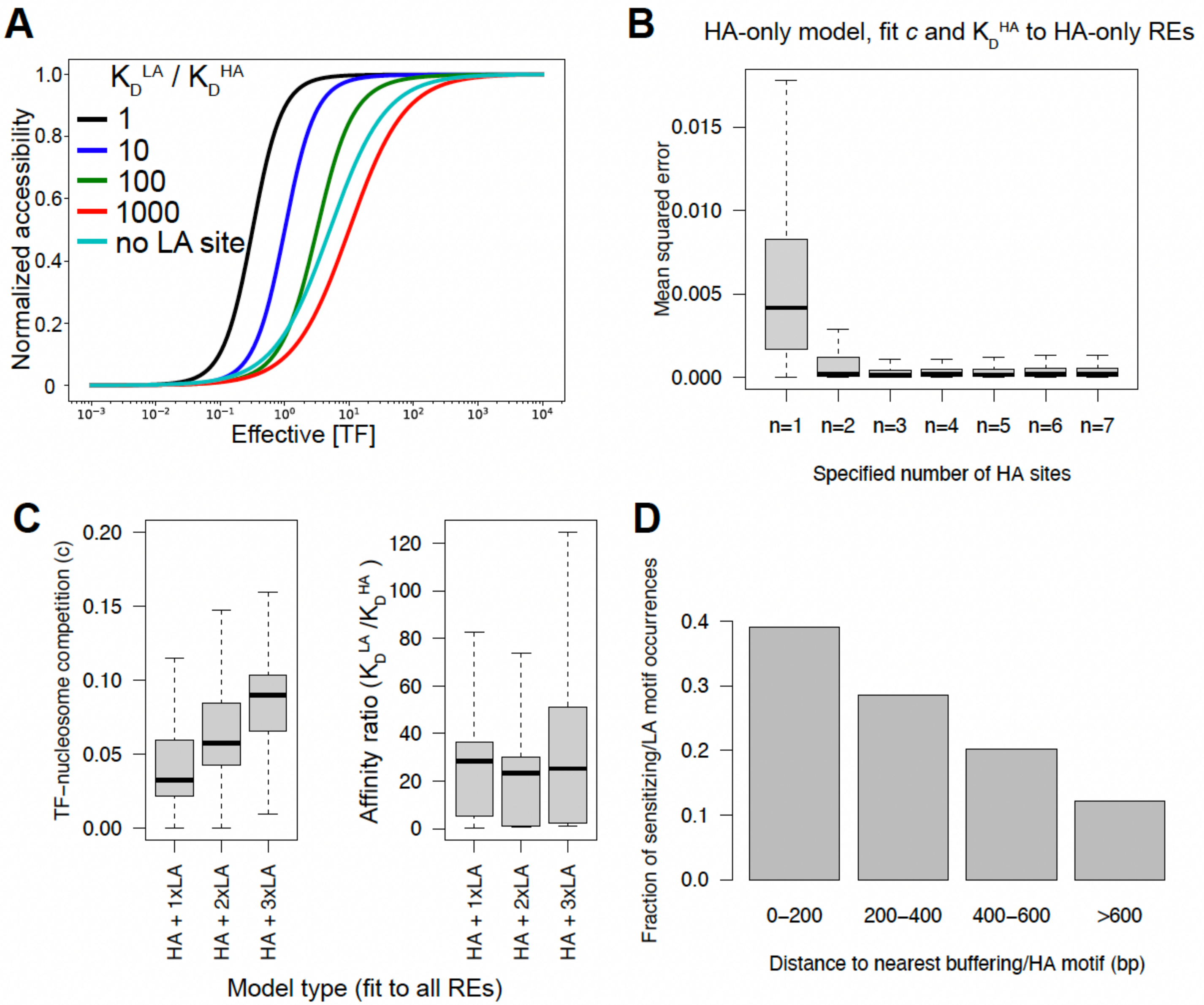

Figure S8. Theoretical and fitted instances of TF-nucleosome competition model. (A) Effect of low-affinity site (colors) or no site (grey) on theoretical dosage curves under model weak TF-nucleosome competition (*c* = 0.001). (B) Mean squared error of high-affinity (HA)-only model with specified effective number of HA sites (x-axis), fit to 1,291 REs. (C) Values of *c* or high/low-affinity site K_D obtained by fitting model to observed dosage response curves for REs a mix of high-and low-affinity Coordinator sites (HA + LA). All REs were fit with the indicated model rather than models matched to the number of LA REs in each. (D) For REs containing a mix of buffering, high-affinity (HA) and sensitizing, low-affinity (LA) motifs, the fraction of LA motifs within the indicated distance to the nearest HA motif is shown.
